# Supplementary material for: Biomechanical Gait Analysis Using a Smartphone-Based Motion Capture System (OpenCap) in Patients with Neurological Disorders
Source: Bioengineering (Basel). 2024 Sep 12;11(9):911. doi: 10.3390/bioengineering11090911 (PMC11429388; doi:10.3390/bioengineering11090911)
Supplement: Supplementary file 1 [file bioengineering-11-00911-s001.zip › bioengineering-3196675-supplementary.pdf]

bioengineering

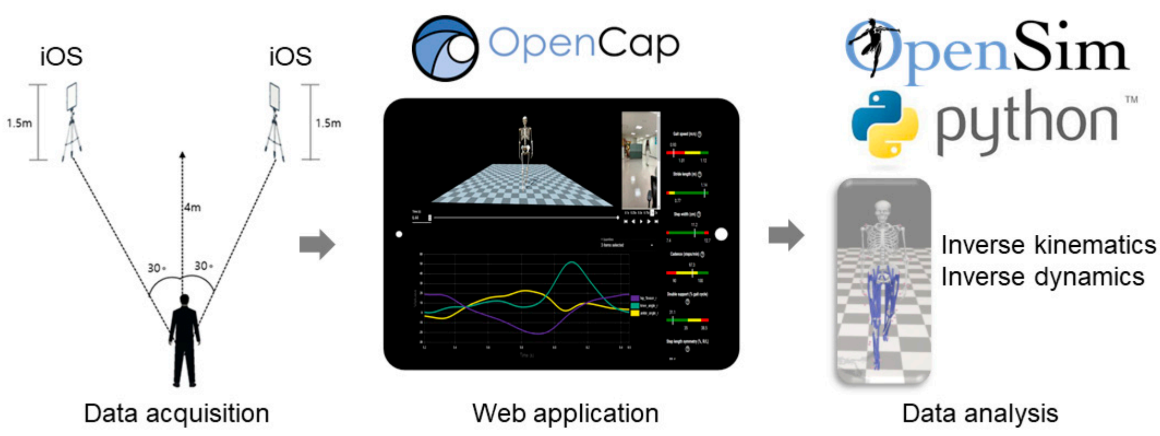

Figure S1. Overview of the Experimental Setup and Data Analysis Workflow. This figure illustrates the comprehensive methodology used in the study, which involves data acquisition using two iOS devices positioned 1.5 meters high and 4 meters apart at a 30-degree angle relative to the subject. The devices are paired with the OpenCap web application, enabling synchronized video recording for multi-view data capture. Spatio-temporal data analysis is then performed in the cloud, where 3D keypoints are extracted from the videos using open-source pose estimation algorithms. Finally, OpenSim and python is employed to perform inverse kinematics and inverse dynamics calculations, using a musculoskeletal model with biomechanical constraints to estimate dynamic measures through muscle-driven simulations that track the 3D kinematics. This workflow integrates smartphone applications, web-based processing, and cloud computing to capture, process, and analyze complex motion data.

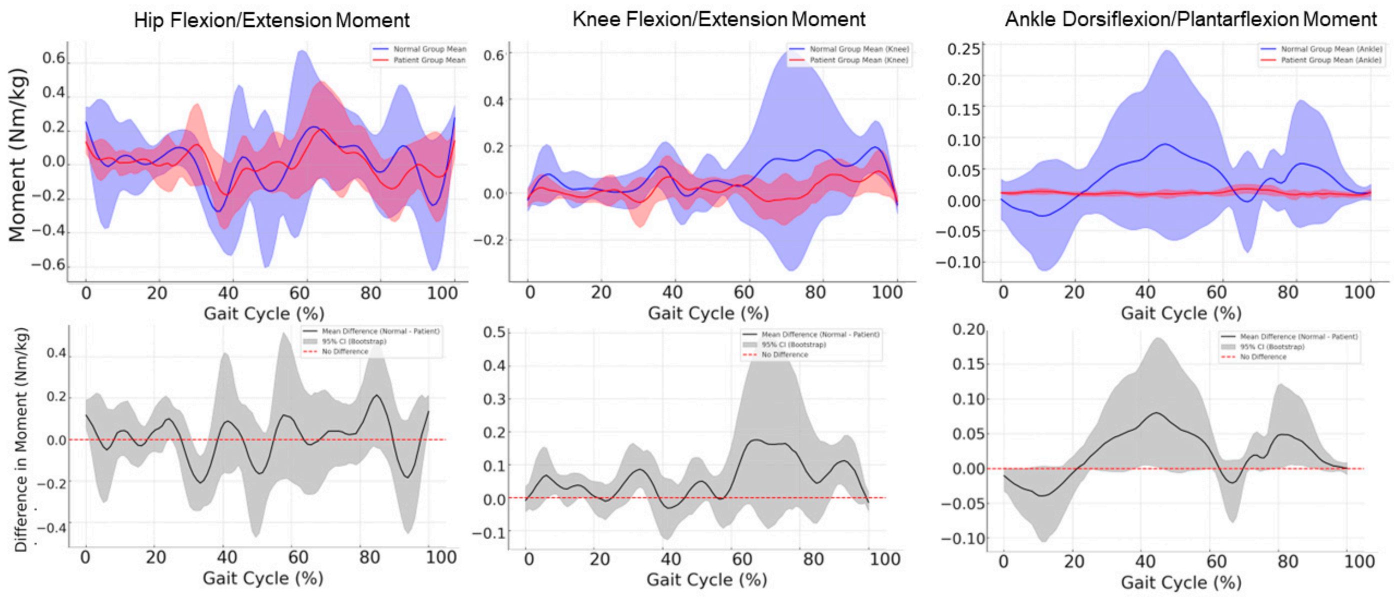

Figure S2. Joint moments during the gait cycle normalized to body weight for the control and patient groups. The top row depicts the mean joint moments for the hip flexion/extension, knee flexion/extension, and ankle dorsiflexion/plantarflexion across the entire gait cycle (%), with separate lines representing the control (blue) and patient (red) groups. Shaded regions represent the ±1 standard deviation (SD) for each group. The bottom row illustrates the mean difference in joint moments between the control and patient groups, with shaded regions showing the 95% bootstrap confidence intervals. Areas where the confidence interval does not intersect the zero line indicate statistically significant differences between the groups. These graphs highlight the biomechanical variations in

joint moments during the gait cycle between healthy individuals and those with neurological disorders, emphasizing areas of significant deviation.

Table S1. Comparative Analysis of Kinematic Parameters during Gait Cycle Between Control and Patient Groups.

Copyright: © 2024 by the authors. Licensee MDPI, Basel, Switzerland. This article is an open access article distributed under the terms and conditions of the Creative Commons Attribution (CC BY) license (https://creativecommons.org/licenses/by/4.0/).

Kinematic Parameter	type	Control (Mean ± SD)	Patients (Mean ± SD)	*p*-value
Pelvis tilt	mean	1.23 ± 2.13	-3.29 ± 4.69	0.0162*
	max	6.05 ± 2.75	0.56 ± 5.16	0.0104*
	min	-3.23 ± 1.94	-7.24 ± 4.73	0.0289*
Pelvis list	mean	-1.61 ± 2.68	-1.39 ± 1.38	0.8256
	max	4.92 ± 1.11	3.40 ± 1.91	0.0458*
	min	-7.67 ± 3.59	-6.58 ± 2.00	0.4157
Pelvis rotation	mean	1.99 ± 3.06	0.10 ± 2.85	0.1689
	max	8.52 ± 3.69	7.27 ± 4.99	0.5360
	min	-4.43 ± 2.32	-5.80 ± 2.98	0.2685
Hip flexion	mean	2.51 ± 3.37	6.23 ± 6.74	0.1419
	max	24.40 ± 2.12	26.15 ± 10.07	0.6032
	min	-21.74 ± 5.94	-11.52 ± 8.64	0.0072*
Hip adduction	mean	1.73 ± 3.40	0.63 ± 2.31	0.4114
	max	10.60 ± 5.29	8.43 ± 3.07	0.2806
	min	-7.94 ± 2.40	-7.54 ± 2.46	0.7225
Hip rotation	mean	-7.18 ± 4.02	-6.49 ± 5.83	0.7628
	max	-1.18 ± 3.31	1.12 ± 6.21	0.3185
	min	-13.68 ± 5.09	-13.77 ± 6.25	0.9726
Knee angle	mean	18.12 ± 3.86	17.63 ± 6.95	0.8496
	max	61.49 ± 5.70	53.95 ± 12.77	0.1130
	min	1.11 ± 0.98	2.12 ± 2.44	0.2470
Ankle angle	mean	5.31 ± 6.59	4.19 ± 5.43	0.6832
	max	20.42 ± 10.51	16.25 ± 6.26	0.2993
	min	-14.19 ± 13.06	-6.67 ± 8.03	0.1416
Subtalar angle	mean	-1.29 ± 9.37	-1.33 ± 7.26	0.9909
	max	9.87 ± 9.90	8.81 ± 10.10	0.8146
	min	-17.11 ± 7.59	-13.90 ± 9.59	0.4184

The kinematic analysis of the gait cycle revealed significant differences between the control and patient groups across several parameters. Notably, **pelvis tilt** demonstrated a significantly lower mean value in patients compared to controls ($p = 0.0162$), with the maximum and minimum values also showing significant differences ($p = 0.0104$ and $p = 0.0289$, respectively). **Hip flexion** exhibited a significant difference in the minimum value ($p = 0.0072$), while **pelvis list** and **knee angle** showed significant differences in their maximum values ($p = 0.0458$ and $p = 0.0458$, respectively). Other parameters, including **pelvis rotation**, **hip adduction**, **hip rotation**, **ankle angle**, and **subtalar angle**, did not show statistically significant differences between the groups. These findings suggest altered

pelvic and lower limb kinematics in the patient group during the gait cycle, indicating potential compensatory mechanisms or pathological changes associated with their condition.

Disclaimer/Publisher's Note: The statements, opinions and data contained in all publications are solely those of the individual author(s) and contributor(s) and not of MDPI and/or the editor(s). MDPI and/or the editor(s) disclaim responsibility for any injury to people or property resulting from any ideas, methods, instructions or products referred to in the content.
